# The Artificial Feeding of Infants

**Published:** 1900-08-18

**Authors:** 


### THE ARTIFICIAL FEEDING OF INFANTS.

As was but natural at a congress held in a city where life has reached the acme of artificiality, the medical men assembled last week in Paris devoted a considerable amount of time to a discussion of the problem of the artificial feeding of infants. Professor Jacobi, of New York, read a paper on the subject, in which he showed how contradictory was the evidence, and how unfounded were many of the beliefs in regard to the subject, and he claimed that although the digestibility of a certain amount of starchy material by infants had long been persistently denied, such digestibility had been proven to exist, and that this had been shown to be true not only by experience but by experiment. Hence cereal decoctions were the proper diluents by which to reduce the excessive proportion of casein in cow's milk. On the whole, he thought that home-made artificial foods were, for many reasons, preferable to the proprietary foods on the market. Dr. Heubner pointed out that too many of our ideas in regard to the artificial feeding of infants were the outcome of the experience of physicians who had been mostly brought into relation with invalid children, and he urged that the common sense of nurses dealing with healthy babies had always led them to see that nothing could replace the mother's milk so well as the milk of some other animal. Truly there was some difference between them, but no greater difference than existed, for example, between the meat of beef and pork, which could quite well be eaten alternately. He thought that the intestine of the healthy infant was capable of digesting the milk of the cow just as well as the milk of a woman, although the labour, the digestive effort required, might be greater in the former case. The danger in all forms of artificial feeding arose from the risk of infection or decomposition of the food before the child got it, and it was here that the advantage of sterilising such food came in. It was not necessary, however, to boil milk for half an hour, five or ten minutes was quite enough, and, indeed, if the milk were heated for 25 minutes to 65 deg. C. it would be sufficiently sterilised and would not undergo any disagreeable modification in taste or nutritive value. Where the child was not healthy, however, the matter was different, and in such cases it was often the inability to digest the full quantity of fat that was the cause of the difficulty. This could be got over by diluting the milk and adding a sufficiency of sugar, or some other carbo-hydrate might be added, such as powdered biscuits, or the milk might be peptonised, or it might be diluted with skim milk, maltose being added instead of sugar of milk. Many other papers were read on the same and cognate subjects, and among them one by M. G. Variot on the methodical employment of sterilised milk, prepared industrially, for the artificial feeding of infants in large towns. He pointed out that in the transport of fresh milk in such towns it was only with great difficulty that fermentations and injurious alterations of its condition could be avoided. Moreover, when milk had once become impure it was unfit for the nourishment of infants, however completely it might be sterilised afterwards, and, therefore, in regard to great cities, it appeared best that the milk should be sterilised before distribution, and even at the place of its production. If properly carried out, the industrial sterilisation of milk in great factories offered great advantages; the small capacity of the flasks in which it was distributed, and the fact that they were hermetically sealed prevented all attempts at fraud, and the milk remained practically sterile, even after the flasks had been opened to fill the feeding bottles, provided that care were taken to replace the stoppers. The conclusions he arrived at from observations made at the dispensary at Belleville, where, during the past four years, 160,000 litres of milk had been distributed, and more than 800 nurslings, whom he had weighed and inspected week by week, had been reared, might be summed up as follows: (1) Milk sterilised industrially and used from bottles graduated according to the age or the weight of the children presents the same advantages as fresh milk collected in the country and sterilised with apparatus of the Soxhlet type. (2) It is demonstrated that the greater number of nurslings bear this milk in its undiluted condition from the age of two or three months, the excess of proteïd substances appearing less harmful than is generally thought. During the first months it has been sufficient to dilute the milk with a third or a quarter of boiled water, with the addition of a little powdered sugar. (3) Not only have healthy children been reared on this milk with success, but more than 300 infants presenting more or less marked atrophy have been brought up upon it. (4) Out of more than 800 nurslings not a single case of Barlow's disease had been met with, and rickets have been very exceptional. On the other hand, constipation and marked anæmia have not been rare.

